# A trio-binning approach for genome assembly reveals extensive structural variation between two Cannabis cultivars: Punto Rojo and Cherry Pie

**DOI:** 10.1093/g3journal/jkaf286

**Published:** 2025-12-30

**Authors:** Brett Pike, Alexander Kozik, Wilson Terán

**Affiliations:** Biología de Plantas y Sistemas Productivos, Departamento de Biología, Pontificia Universidad Javeriana, Bogotá, Distrito Capital 110231, Colombia; Medicamentos de Cannabis SAS, Bogotá, Distrito Capital 111111, Colombia; Genome Center and Department of Plant Sciences, University of California, Davis, California, CA 95616, United States; Biología de Plantas y Sistemas Productivos, Departamento de Biología, Pontificia Universidad Javeriana, Bogotá, Distrito Capital 110231, Colombia

**Keywords:** genome assembly, Oxford Nanopore, trio binning, Cannabis, terpene synthases, NLR genes

## Abstract

With the advent of long-read DNA sequencing technologies, assembling eukaryotic genomes has become routine; however, properly phasing the maternal and paternal contributions, which is of great value for breeding programs, remains technically challenging. Here, we use the trio-binning approach to separate Oxford Nanopore reads derived from a Cannabis F1 wide cross, made between the Colombian landrace Punto Rojo and the Colorado CBD clone Cherry Pie #16. Reads were obtained from a single PromethION flow cell, generating assemblies with coverage of just 18 × per haplotype, but with good contiguity and gene completeness, demonstrating that it is a cost-effective approach for genome-wide and high-quality haplotype phasing. Evaluated through the lenses of disease resistance and secondary metabolite synthesis, both being traits of interest for the Cannabis industry, we report copy number and structural variation that, as has recently been shown for other major crops, may contribute to phenotypic variation along several relevant dimensions.

## Introduction

Cannabis is a dioecious annual crop, and its closest relative is *Humulus*, a genus of three species whose most famous member is *H. lupus*, or brewers' hops. Divergence from their common ancestor is thought to have taken place about 28 MYA in what is today northeast Tibet (McPartland et al. 2019). Cannabis landraces spread to Southeast and Southwest Asia (Ren et al. 2021), and later, among other dispersals, to Africa and then South America (Warf 2014).

Following 100 years of prohibition, Cannabis is again legal in many countries and jurisdictions, driven by its growing acceptance and awareness of its potential therapeutic benefits. This has boosted cannabis research and given rise to the medical cannabis industry, with a market valued at $21.4 billion for 2025, expected to surpass $200 B in the next decade (Metatech Insights 2024). Despite the economic and cultural importance of Cannabis, it is notable that genetic resources are scant (Kovalchuk et al. 2020), highlighting also the need for modern breeding programs to accompany this global market growth. Cannabis genomics has, therefore, appeared as an emerging topic to fill the lack of genetic knowledge.

The first Cannabis genome to be anointed as the reference by NCBI, a CBD type from Colorado called cs10 (Grassa et al. 2021), offers good contiguity and genic content, and so we have used it as the primary point of comparison in our analyses. However, as a collapsed pseudohaploid, its scaffolds cannot represent the true range of variation found within an individual, and as a modern polyhybrid, it cannot inform as to the ancestral state of the Cannabis population's founders. In an effort to address this lacuna, we have sequenced an F_1_ derived from two distantly related parents, which vary for several agronomic traits of interest: height, flowering time, cannabinoid content, terpene content, and fungal susceptibility.

To facilitate comparative genomics and establish a genome-wide resource for trait mapping and marker development, we assembled both haplotypes of this wide cross via trio-binning of Oxford Nanopore reads. This approach allowed us to obtain fully phased chromosome-scale assemblies with good contiguity and gene completeness, which provide accurate catalogs of important gene families, specifically disease resistance genes of the Nucleotide-binding, Leucine-rich Repeat type (NLRs) and terpene synthases (TPS).

## Materials and methods

### Breeding materials

The sequenced individual was an F_1_ hybrid between the psychoactive Colombian landrace “Punto Rojo #3” (PR) and the nonpsychoactive Coloradan line “Cherry Pie #16” (CP). Both parental clones have been formally characterized and registered with the Instituto Colombiano Agropecuario (ICA) by Medicamentos de Cannabis SAS.

**Fig. 1.**
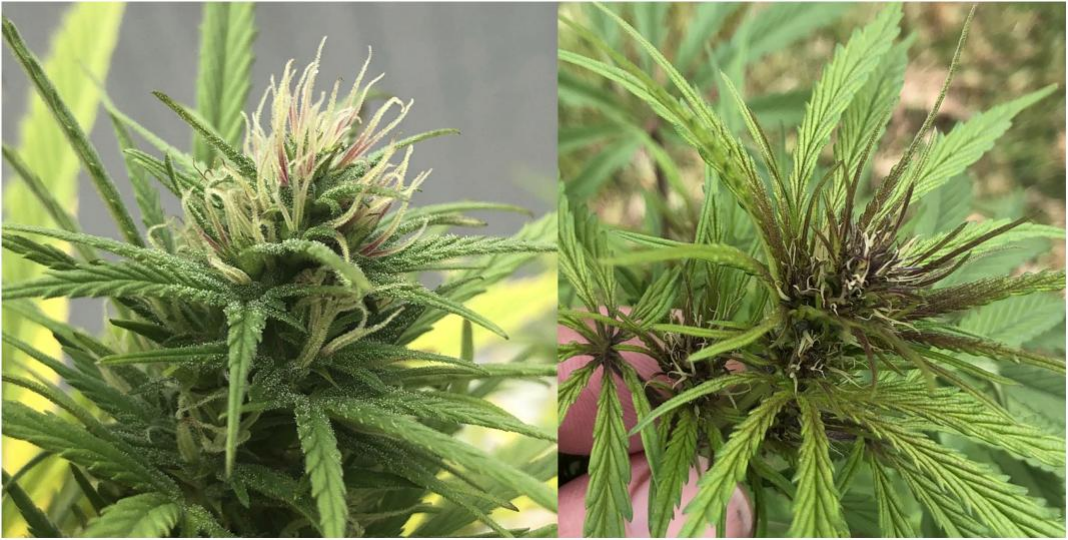
The Punto Rojo phenotype may describe anthocyanin deposition in the calyxes (left) or new shoots (right). Photos by Brett Pike.

Punto Rojo is thought to descend from dual-use (drug and fiber) African cannabis introduced to Colombia in the 17th century (Warf 2014), and has acclimatized almost entirely in the absence of irrigation, fertilization, and agrochemicals. It has good resistance to fungi and grows well in high heat and low-nutrient soil. The name translates as “Red Point” and refers to the unusual levels of anthocyanin sometimes seen in new shoots and receptive calyces (Fig. 1). In the 60s and 70s, illicit shipments of Type I (THC-dominant) Punto Rojo found favor among American consumers due to its special effects, which were thought to be more psychedelic and less soporific than other imports (Kala 2021).

**Fig. 2.**
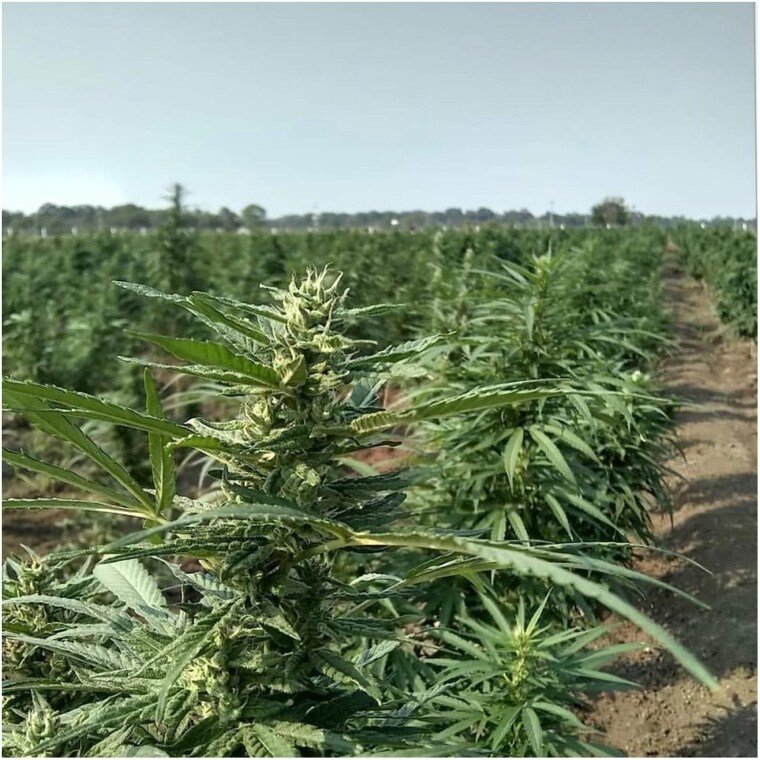
Clones of Cherry Pie #16 flowering in Fuente de Oro, Meta, Colombia. Photo courtesy of Medcann Pharma.

Cherry Pie (Fig. 2) is one of several Type III (CBD-dominant) strains in the Cherry family, bred in the American state of Colorado following legalization. Cherry Blossom (Anderson et al. 2021) and Cherry Wine (DiMatteo et al. 2020) have been the subjects of recent reports, and the initial NCBI reference for Cannabis, CBDRx (Grassa et al. 2021), falls into this clade as well. All display fast flowering and high CBD content, as well as a pleasant cherry aroma. The CP-16 individual was selected for consistently containing less than 1% THC at maturity, which enables its registration as non-psychoactive under Colombian law. This permits unlimited cultivation for any licensed cultivator, without diminishing Colombia's share of the global THC quota established by the United Nations Office on Drugs and Crime.

### Plant growth

The F_0_s and the F_1_ were grown at the licensed farm of Medicamentos de Cannabis SAS near Fuente de Oro, Meta, Colombia, as approved by the Ministry of Justice in Resolution 1164 of August 19, 2021. At this latitude (3.47° N), the photoperiod is consistently 12 h, and therefore always inductive for Cannabis flowering. At this altitude (400 m), the average day and night temperatures are 30 °C and 21 °C. The F_0_ clones had previously been selected from seed and then propagated clonally.

Clones were rooted in Oasis-type plugs under fluorescent lamps and then transplanted to 15 L containers filled with 70% coco fiber and 30% worm castings, watered by hand, in a trailer about 2 m × 4 m, fitted with 2 1,000 W HPS lamps and an air conditioner set to 16C. The CP-16 female was induced to produce female (XX) pollen via two applications of 0.03% silver nitrate, at 0 and 7 d of flowering, which was then blown towards a group of females, including PR, with the aid of an oscillating fan. F_1_ seeds were sown in two 144-cell trays and, after 21 d, 250 seedlings were transplanted to 3 L containers filled with a mix of 70% coco fiber and 30% worm castings. These plants grew vegetatively for a total of 60 d with 12 h of sunlight and supplemental lighting from 6 pm to midnight. They were next transplanted to the field at a density of 2 plants per square meter into holes amended with one handful of a mix consisting of 50% worm castings, 20% rock phosphate, 20% dolomite lime, and 10% Peruvian bat guano. The plants were rain-fed, with additional watering by hand as needed.

About 40 d after transplant to the natural inductive photoperiod, an individual (PC-67) was chosen that was approximately average for the population in terms of height, flower development, leaf morphology, internode spacing, and degree of branching. As well, its flowers produced an aroma that evoked both the red fruit odor of Cherry Pie and the citric tanginess of Punto Rojo. PC-67 was cloned and propagated vegetatively, and about 12 wk later, new shoots consisting primarily of unexpanded leaves were sampled for DNA sequencing.

### DNA purification

DNA from the F_0_s was extracted from new shoots dried over silica with a Quick-DNA Plant/Seed Miniprep Kit (Zymo Research, Irvine, California, USA). For the F_1_, HMW DNA was purified from clean nuclei as described previously (Pike et al. 2021) and then size-selected via the Short Read Eliminator XL kit (Circulomics, Baltimore, Maryland, USA). Several replicates were combined to yield a sufficient quantity. DNA concentration and purity were estimated through the use of NanoDrop 1000 (Thermo Scientific), and two additional ethanol washes on SPRI beads were performed to meet sequencing standards.

### F_0_ Illumina library prep and sequencing

The F_0_s were prepared as Illumina TruSeq libraries and sequenced as part of a NovaSeq PE150 lane. Illumina reads were filtered with BBDuk (Bushnell 2018) to remove adapter sequences low-quality reads, and short reads using default parameters. These reads were filtered against Cannabis chloroplast and mitochondrial genomes using CLC Genomics Workbench, for subsequent assembly into contigs. The resulting sequences were used as BLAST queries, using MegaBLAST with default parameters in Geneious Prime, against a custom database comprising the genomes of seven fungi known or suspected to be present in the field: *Aspergillus fumigatus*, *Botrytis cinerea*, *Cercospora beticola*, *Fusarium oxysporum*, *Pseudocercospora fijiensis*, *Pseudocercospora musae*, and *Sclerotinia sclerotiorum*. Following these results, reads were then mapped with BBSplit (Bushnell 2018) to the genomes of *P. fijiensis* and *P. musae*, as the final filtering step in order to remove these contaminating sequences.

### F_1_ Oxford Nanopore library prep and sequencing

The HMW sample was analyzed for length distribution via Agilent Femto-Pulse. Then, an Oxford Nanopore library was prepared (ligation kit LSK-0110) and sequenced in one PromethION R9.4.1 cell. After 24 h, a nuclease flush was performed, and the library was then reloaded and sequenced for another 72 h. Basecalling was performed by Guppy 5.1.12 in “super-accurate” mode.

All library preparation and sequencing took place at The Genome Center at the University of California, Davis.

### Genome assemblies

Precise syntax for each command may be found at https://github.com/COMInterop/PRCP. Specific versions of programs used are listed in Supplementary Table 6.

#### Genome size estimation

21-mers were counted in both sets of F_0_ short reads with jellyfish (Marçais and Kingsford 2011) and histograms evaluated with findGSE (Sun et al. 2018) in homozygous and heterozygous mode, with the latter using expected homozygous coverage of 18 (exp_hom = 18). This process was repeated with the binned, error-corrected F_1_ long reads.

#### Assembly

Trio binning was performed with scripts written for the purpose (Rice 2019). Briefly, 21-mers were counted with KMC (Kokot et al. 2017), unique parental 21-mers were derived by “find-unique-kmers,” and 21-mers containing homopentamer repeats were deleted with a simple *grep* command. These lists were then used with “classify_by_kmers” to sort long reads into PR, CP, and unknown bins.

Binned reads were assembled into contigs with NECAT (Chen et al. 2021), and the unbinned reads were ignored. Assembly included all reads longer than 3 kb with the default parameters and “polish contigs = false”. Contigs identified as mitochondrial by NCBI were removed. Assembly transpired on an AWS EC2 “m6gd.metal” instance, with 64 ARM cores and 256 Gb RAM.

#### Polishing

Each haplotype's binned raw reads were filtered for quality at 7 and aligned to their assembly with Minimap2 (Li 2021), with options “-aL -z 600,200 -x map-ont”. One round of polishing then took place with Racon (Vaser et al. 2017) with the “-u” option. Next, the appropriate F_0_ short reads were mapped to each haplotype with BWA MEM (Li and Durbin 2009) and polished with Clair3, twice. In the first round, Clair3 used the options “–haploid_precise –no_phasing_for_fa,” which only generates well-supported 1/1 calls. In the second round, all variants were called: 0/1 calls were deleted, 1/1 calls were applied, and where possible the shorter allele in 1/2 calls was applied with the command “bcftools consensus -H SR” Finally, each assembly was polished 4 times with its F_0_ kmers with ntEdit (Warren et al. 2019), using default settings and kmer lengths of 40, 26, 40, and 26.

Polishing and other post-assembly processing took place on a 2012 Mac Pro 5,1 with 2 Xeon X5690 processors and 64 Gb RAM.

#### Scaffolding

Scaffolding was performed with ntJoin (Coombe et al. 2020) with options “nocut = True” and “overlap = False,” and a maximum gap of 100,000 bp. The substrate was derived from the Salk Institute's recent release of many phased haplotypes (Lynch et al. 2025), which was subsetted to include 8 drug haplotypes assembled with the benefit of Hi-C libraries. PR and CP contigs were first aligned to each haplotype, and alignments were inspected visually with dotplotly (Poorten 2017). For each chromosome, the homolog with the most diagonal alignment was chosen. Then, a small number of additional substitutions were made to reduce interchromosomal translocations. The superscaffolds ultimately used for each genotype are listed in Supplementary Table 1.

Finally, the chromosome-scale pseudomolecules were aligned to the cs10 reference genome and, where necessary, reverse complemented to maintain a consistent orientation. For PR, chromosomes 2, 3, 4, 8, and 9 were reversed, and for CP, 1, 6, 7, 8, 9, and X.

#### Evaluation

Assemblies were evaluated for contiguity with the BBTools script stats.sh (Bushnell 2018), for completeness with compleasm (Huang and Li 2023) using the eudicots_odb10 5.4.6 database, and for correctness, including phasing accuracy, with Merqury, after counting 20-mers in F_0_ short reads and error-corrected F_1_ long reads with Meryl (Rhie et al. 2020).

Analysis of the contigs' long-read coverage was performed with Flagger (Liao et al. 2023). Assemblies were screened with “yak qv”, and high-error-rate subsequences (HERS, Chen et al. 2021), here defined as the basespace unable to be verified by comparison with short-read 21-mers, were compiled and exported as a BED.

#### Organelles

F_0_ short reads identified as organellar were mapped to the Yunma-7 chloroplast and Carmagnola mitochondrion with the Geneious Prime 2023.0.4 mapper using default settings. The consensus sequences for each were generated and appended to each long-read assembly.

#### Diploid assembly

To test diploid-aware assembly methods, drafts were assembled in PECAT (Nie et al. 2024) and Shasta (Lorig-Roach et al. 2023). PECAT used the configuration for Arabidopsis (cfg_arab_ont) with some modifications. Briefly, PECAT's block size for correction and assembly, and Minimap2's index and minibatch size, were raised to 40 Gb to enable true all-vs-all alignment; for correction, minimum coverage was lowered to 2 for correcting and to 8 for calling SNPs for haplotypes; for assembly, the contig duplication rate was set to zero and only contigs over 4 kb were outputted; and for phasing, minimum coverage was lowered to 16. The primary assembly was purged of haplotigs with purge_haplotigs (Roach et al. 2018), and the purgate was combined with the alternate assembly, which was purged a second time.

Shasta used the Nanopore-Phased-May2022 configuration, and its output was further processed to resolve haplotypes: Assembly-Detailed.gfa and parental 31-mer databases generated with KMC (Kokot et al. 2017) were analyzed with GFAse (Lorig-Roach et al. 2023) to produce unphased, maternal, and paternal FASTAs.

Haplotype resolution at the contig level was visualized with Merqury. These assemblies went unpolished, and so QV is not reported. Contiguity and completeness were measured as above.

Diploid-aware analyses were performed on the “pyky” node of the ZINE high-performance compute cluster at the Pontificia Universidad Javeriana, which includes 192 CPUs and 2 Tb of RAM.

### Annotation

#### Whole genome

Gene annotations were transferred from the cs10 reference to these drafts with Liftoff (Shumate and Salzberg 2021), with options “-f features.txt -chroms chroms.txt -copies -sc 0.99,” where features.txt includes all annotation types except “regions”, chroms.txt lists the most likely homolog for each pseudomolecule, based on a preliminary synteny analysis with SyRI (Goel et al. 2019), and “-copies -sc 0.99” seeks to find paralogs that have at least 99% exonic identity to the primary annotation.

#### Cannabinoid synthases

Cannabinoid synthases were predicted *ab initio* in the assemblies listed in Table 2 by using the “Annotate From…” function in Geneious Prime 2023.0.4 (https://www.geneious.com), using the full-length CDS for either THCAS from Skunk #1 (Weiblen et al. 2015) or the 6-3 allele of CBDAS (Onofri et al. 2015), a similarity threshold of 85%, and the “All matching annotations” option. Gene clusters were then visualized in Geneious Prime.

#### Terpene synthases

The cs10 annotations were filtered for the presence of the following descriptive terms: farnesene, geraniol, germacrene, humulene, limonene, linalool, myrcene, nerolidol, pinene, terpene, terpenoid, or terpinolene. The 47 annotations thus labelled were then transferred with Liftoff to both drafts, with stringency relaxed via “-copies -sc 0.50,” to locate any additional paralogs that have similar structure and share at least 50% exonic identity. To predict products, a custom BLAST database was built in Geneious Prime 2025.1.2 using the amino acid sequences of 33 TPS characterized via heterologous expression (Booth et al. 2020). Predicted TPS were queried against this database with blastx, and in some cases, multiply aligned with Clustal Omega (Sievers and Higgins 2014).

#### NLR genes

The NBS_712 HMM (Kozik 2001), which covers the highly conserved nucleotide binding site (NBS) region of NLRs and was initially derived from the Arabidopsis genome(Meyers et al. 2003), was queried with BLAST against the cs10 reference to create an initial list of candidates. These regions were extracted, aligned with Clustal Omega, and used to create a Cannabis-specific NBS Hidden Markov Model (CsNBS HMM) via the hmmbuild and hmmemit modalities of the HMMER (Finn et al. 2011) software package. The DNA consensus of the HMM was then BLASTed against the PR and CP drafts, and hits, after merging overlaps, were taken as putative NLR loci. As well, the NLR-Annotator (Steuernagel et al. 2020) was used to make a set of predictions, and the intersection of the two callsets was taken, so that full-length gene predictions from NLR-Annotator, verified by CsNBS HMM hits, are reported.

#### Repetitive elements

Each haplotype was analyzed with EDTA, the Extensive de novo Transposable element Annotator (Ou et al. 2019), with setting “–force 1 –sensitive 1 –anno 1,” and incorporating the CDS from cs10 to avoid calling genes as repeats. The LTR Assembly Index (LAI, Ou et al. 2018) was calculated from the EDTA output.

### Comparison

Drafts of PR and CP were each scaffolded to and then aligned against the collection of pseudomolecules listed in Supplementary Table 1. Alignments were performed with Minimap2 with options “-cx asm5 –cs –eqx” and visualized as a dotplot with dotplotly (Poorten 2017). The resultant PAFs were analyzed with SyRI (Goel et al. 2019) with default options, and visualized as a synteny map by plotting the SyRI calls with plotsr (Goel and Schneeberger 2022). The PR and CP haplotypes were also compared to each other, and visualized in Circos (Krzywinski et al. 2009). The two assemblies, along with the genomes used for scaffolding and the current and prior NCBI references, were analyzed with ntSynt (Coombe et al. 2024) with a minimum block size of 100 kb, and visualized with ntSynt-viz (Coombe et al. 2025), with PR specified as the target genome.

## Results

### HMW gDNA

Each prep of one gram of young shoots provided about 4 μg high-quality DNA, with 260/280 of 1.8 and 260/230 of 2.0. Analysis via Agilent Femto-Pulse showed that this method retains many fragments over 100 kb, and the steep decline in fragments below ∼19 kb suggests that the Short Read Eliminator XL kit did function as advertised (Supplementary Fig. 1).

### F_0_ Illumina sequencing

The Illumina libraries yielded 52.6 M and 47.6 M read pairs for PR and CP. Filtering the reads resulted in sets mapping to the chloroplast and mitochondria, as well as to two species of *Pseudocercospora*. Compared to a reference mitochondrion from the hemp line Carmagnola, PR contained 197 SNPs and CP 80. Compared to a reference chloroplast from Yunma-7, PR contained 9 SNPs and CP 125. About 0.5% of reads mapped to *Pseudocercospora*, with *P. musae* appearing to be about 50% more abundant than *P. fijiensis* in both F_0_s (data not shown). After trimming and decontamination, 85.0 and 81.1% of base space remained, providing 16.7 × and 14.4 × of coverage for polishing.

### F_1_ Oxford Nanopore sequencing

The PromethION cell yielded 34.6 Gb of data, with an N_50_ of 23.6 kb, and 15.5% of bases contained in reads over 50 kb.

### Estimation of genome size

Estimates of genome size were derived from both short and binned, corrected long reads. Results from findGSE are summarized in Table 1.

**Table 1.** Genome size estimates in Mb, derived from F_0_ short and binned, corrected F_1_ long reads.

readset	findGSE (hom)	error-excluded	findGSE (het)	error-excluded
PR-ilmn	857.661	823.229	857.661	819.607
PR-ONT-bin-corr	827.774	784.051	fail	fail
CP-ilmn	78.037	42.676	994.974	955.885
CP-ONT-bin-corr	794.321	751.697	fail	fail

### Assembly

Assembly statistics, including the two drafts presented here, a recent NCBI upload, and three previously published chromosome-scale long-read assemblies (McKernan et al. 2018; Gao et al. 2020; Grassa et al. 2021), are summarized in Table 2.

**Table 2.** Assembly statistics for PR, CP, and other Cannabis chromosome scale assemblies published since 2020.

Genotype	Punto Rojo	Cherry Pie	Abacus	Cannbio-2	cs10	JL
**Reference:**	This study		–	Braich et al 2020	Grassa et al 2021	Gao et al 2020
**GenBank**	JBDLLE000000000	JBDLLD000000000	GCA_025232715.1	GCA_016165845.1	GCA_900626175.2	GCA_013030365.1
**Long read platform**	ONT		PacBio Sequel	PacBio SMRT	ONT	PacBio Sequel
**Long read coverage**	18x		83×	86×	36×	**153×**
**Short read platform**	pe150		pe250	–	pe150	pe150
**Short read coverage**	18x		**234**×	–	100×	118×
**Assembler**	NECAT		unk.	HGAP4	miniasm	Wtdbg, SMARTdenovo, Quickmerge
**Long read polishing**	Racon		unk.	HGAP4	3 × Racon	blasr, Arrow
**Short read polishing**	2 × Clair3, 4 × ntEdit		unk.	–	3 × Pilon	Pilon
**Scaffolder**	ntJoin		Hi-C	RaGOO	Hi-C	Hi-C
**Scaffold reference**	Salk assortment (Supplementary Table 1)		de novo	cs10	de novo	de novo
**Linkage map**	–		–	–	Skunk × Carmen	–
**Haplotyping**	trio-bin		unk.	–	–	–
**# contigs**	867	1,171	1,023	8,919	**831**	2,978
**contig size (Mb)**	740	724	796	914	714	812
**contig N50 (Mb)**	2.12	1.65	**3.17**	0.17	2.14	0.51
**contig N90 (Kb)**	413	349	343	46	**459**	126
**contig max (Mb)**	9.84	7.86	**16.96**	1.56	10.06	2.87
**# scaffolds**	**10**	**10**	160	147	**10**	483
**scaffold size (Mb)**	794	774	797	914	854	813
**scaffold N50 (Mb)**	87.6	81.1	80.6	91.5	91.9	83.0
**scaffold N90 (Mb)**	66.4	68.9	63.0	71.6	64.6	69.1
***N* %**	6.71%	6.40%	0.01%	0.10%	16.34%	0.09%
**BUSCO score (% single)**	**98.6%**	94.5%	97.2%	97.1%	94.0%	92.8%
**BUSCO single**	**2,173**	2,149	2,008	1,373	2,031	1,794
**BUSCO duplicated**	121	**49**	252	886	156	364
**BUSCO fragmented**	**7**	21	9	16	16	23
**BUSCO missing**	**25**	107	57	51	123	145
**Merqury Quality Value (QV)**	24.41	24.38				

High marks are bolded where relevant. All BUSCO scores were newly calculated in compleasm with the eudicots_odb10 5.4.6 database.

#### Trio binning

The “classify_by_kmers” script produced a PR bin containing 17,605 Mb of sequence in 1,238,187 reads, and a CP bin containing 15,942 Mb of sequence in 1,156,998 reads. The unknown bin contained 889 Mb in 322,224 reads, which did not assemble into contigs and were not analyzed further. The split among PR, CP, and unknown was 51.1%, 46.3%, and 2.6%. After assembly and polishing, the switch rates for PR and CP were estimated by Merqury as 1.00 and 0.62%, kmer completeness was 97.81 and 98.25%, and the content of other-parent hampers was 0.29 and 0.32%.

#### Contiguity

The drafts of Punto Rojo and Cherry Pie contain 867 and 1,171 contigs, with N_50_ of 2.12 and 1.65 Mb, N_90_ of 413 and 349 Kb, and a longest contig of 9.84 and 7.86 Mb.

We verified the integrity of the contigs with Flagger, which identified 66 and 178 potential error regions in PR and CP (Supplementary Table 2 and *-flagger.bed annotations), of which 64 and 177 were at the ends of contigs, where a drop in coverage is not unexpected. To evaluate the 3 intra-contig error regions, of which one contained one gene and two were non-genic, we ran BLAST queries with the closest genes on either side, which confirmed that gene order was conserved (relative to ERBxHO40_23, data not shown), and so we have elected to leave them in their original state.

Scaffolding PR and CP with ntJoin resulted in placement of 97.7 and 96.4% of contig sequence on the 10 chromosome-scale pseudomolecules, with N content of 6.71 and 6.40%.

#### Completeness

**Fig. 3.**
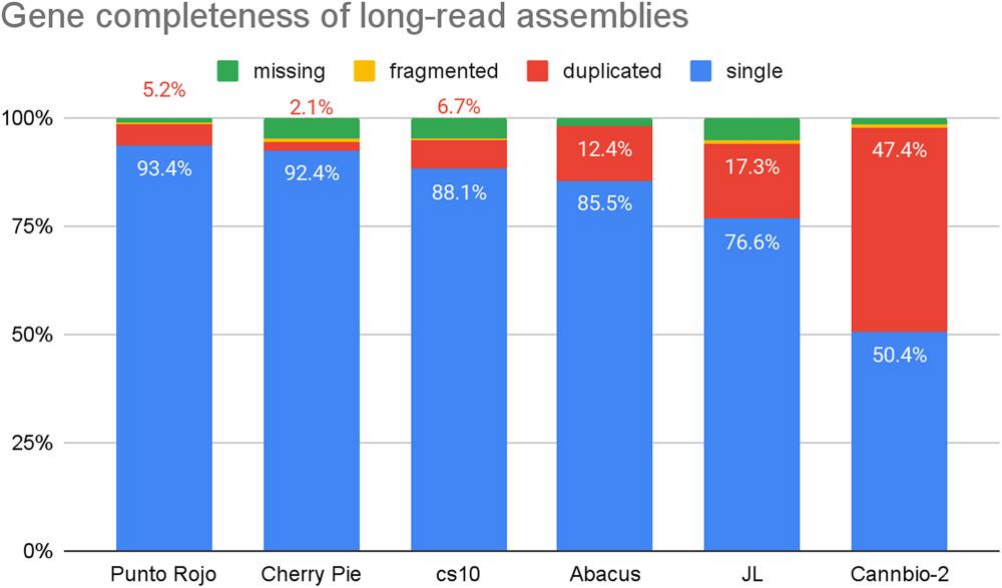
Completeness of long-read Cannabis assemblies. BUSCO scores are expressed in percent of total plant orthologs, with different colour labels for single, duplicated, fragmented and missing genes. Previously published assemblies were newly evaluated with the eudicots_odb10 5.4.6 dataset.

PR and CP have compleasm BUSCO scores of 98.6 and 94.5%, with duplication ratios of 5.2 and 2.1%. The full BUSCO output is summarized in Table 2 and Fig. 3.

#### Correctness

For PR and CP, Merqury estimates QV at 24.42 and 24.35, corresponding to base level precision of 99.64 and 99.63%. Yak QV annotated 19.85 Mb and 21.67 Mb in 801k and 884k high-error-rare subsequences, with the large majority of HERS (736k and 815k) being under 50 bp (*-yak-hers.bed annotations), and just 1 and 2 being over 1 kb.

#### Diploid assembly

PECAT + purge_haplotigs produced a primary and an alternate assembly. Shasta produced a diploid draft, which was subsequently binned by GFAse into maternal, paternal, and unknown compartments. The size, contiguity, and completeness are reported in Table 3.

**Table 3.** Assembly statistics for trio-binning, PECAT, Shasta, and Shasta + GFAse using F_0_ kmers.

	Total size (Mb)	Contigs	N50 (Kb)	BUSCO-total	BUSCO-duplicate
triobin_pr	740	867	2,120	98.60%	5.60%
triobin_cp	724	1,171	1,650	94.50%	2.90%
PECAT_pri	723	464	2,336	96.70%	4.47%
PECAT_alt	717	1,556	908	90.70%	3.57%
Shasta-dip	877	742,931	65	94.24%	60.20%
GFAse-mat	435	3,249	234	83.87%	1.07%
GFAse-pat	439	3,250	235	83.88%	0.86%
GFAse-unphased	272	24,384	51	16.25%	4.43%

**Fig. 4.**
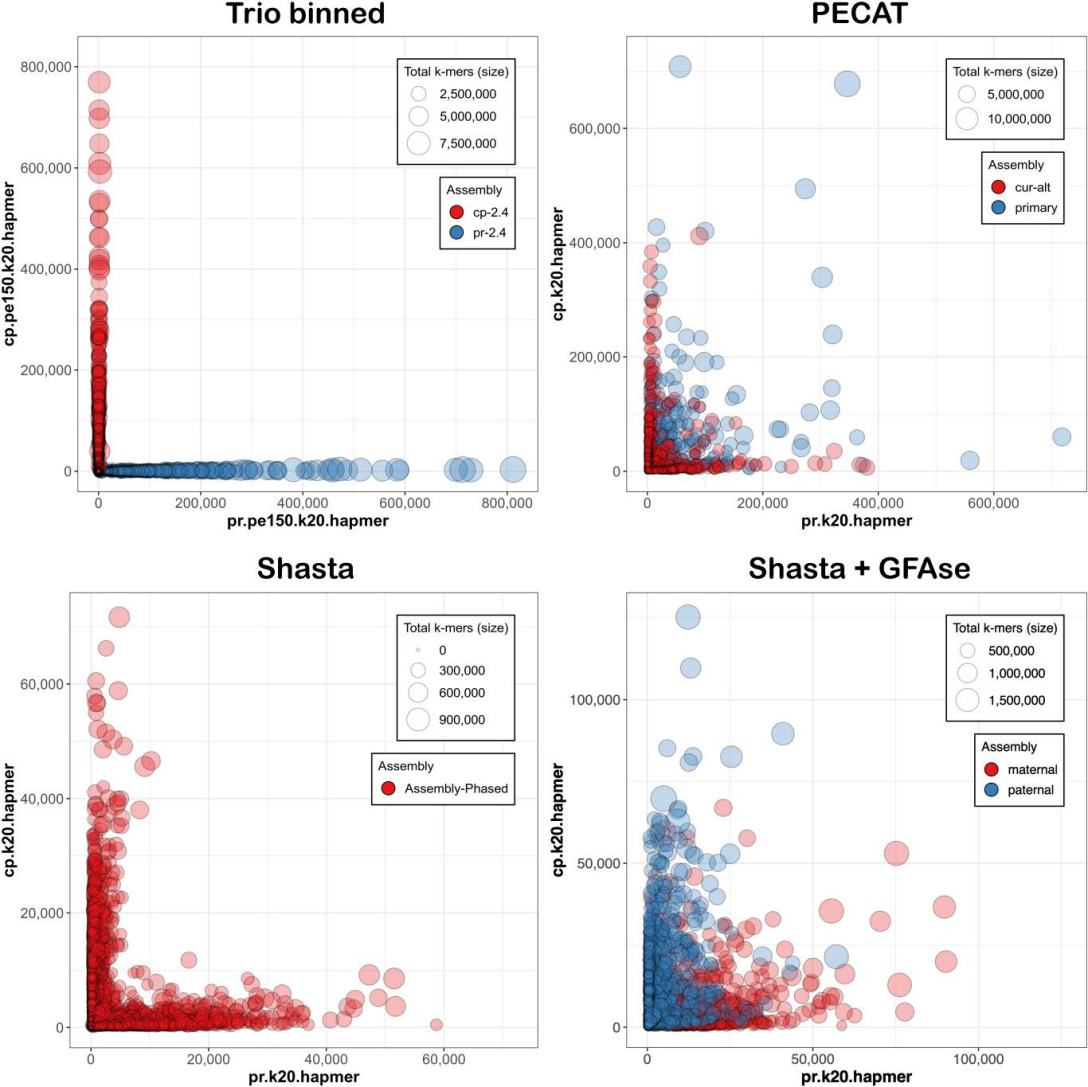
Merqury plots, where the *X* and *Y* axis represent the number of unique PR and CP 20-mers. Please note that scaling varies among drafts.

Haplotype separation was visualized in Merqury, based on per-contig counts of parental short-read 20-mers as tabulated by Meryl (Fig. 4).

### Annotation

#### Liftoff

Nearly all of the reference annotations were able to be placed on both drafts. Table 4 summarizes the drafts' annotations.

**Table 4.** Accounting of cs10 annotations transferred to PR and CP with Liftoff, with option “-copies 0.99”.

	cs10	CP	CP (%)	PR	PR (%)
gene	29,807	29,008	97.32%	29,851	100.15%
pseudogene	1,363	783	57.45%	824	60.45%
mRNA	33,639	31,804	94.55%	32,854	97.67%
CDS	33,674	31,734	94.24%	32,823	97.47%

#### CN synthases

**Fig. 5.**
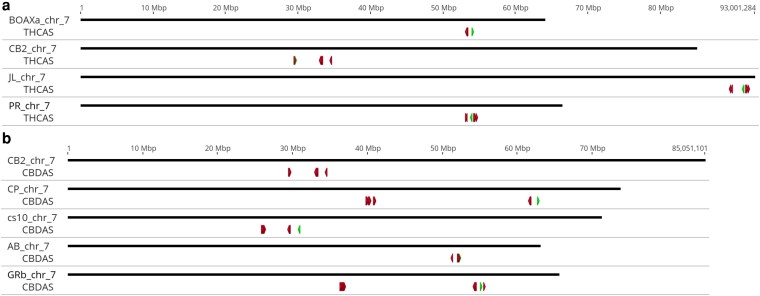
Visualization of the a) Bt and b) Bd alleles on chromosome 7 from the published assemblies and the haplotypes used for scaffolding (BOAXa for PR and GRb for CP). The active synthase is marked in green, while inactive paralogs are in red.

The primary location for CN synthases, which includes 6 to 13 paralogs with identity from 85.3 to 99.9%, is the previously identified B locus (de Meijer et al. 2003; Grassa et al. 2021) on chr7, which varies in size, location, and copy number among assemblies (Supplementary Table 4 and Fig. 5). We note here that JL numbers its chromosomes in order of length, so that its chr1 is the homolog of chr7 in cs10 and the other listed assemblies. Because PR and JL do not include a CBDAS above 95% identity, and CP and Abacus do not include a THCAS above 95% identity, we report only the relevant CN synthase query and homology scores for paralogs of the putative active gene, which in all cases shares >99% identity with the query. However, we note that in no case does a query with the other CN synthase return a different copy number (data not shown). Because Cannbio-2 is a pseudohaploid representation of a B_D_/B_T_ genotype, its results are reported for both queries.

The arrangement of CBDAS copies appears to offer more variability. While most drafts contain all synthase copies in one cluster of 5 Mb or less, CP has two clusters, both on chr7: a group of 5 containing the active synthase at 61.7 to 62.7 Mb, and a group of 8 paralogs with 88 to 89% identity that spans from 39.8 to 40.9 Mb. The Golden Redwood B haplotype to which it is scaffolded appears similar, but contains 7 and 10 copies in similarly situated clusters.

#### TPS

The annotations transferred from cs10 were mined for descriptions that included the name of a terpene. 45, 41, and 47 TPS were located in PR, CP, and cs10 (Supplementary Table 3). The TPS are unevenly distributed, with clusters of monoterpene or diterpene synthases lying in distal regions of chromosomes 5, 6, and 9. We denote these as Major Terpene Clusters (MTC, Table 5), defined here as a group of at least 4 TPS genes separated from one another by no more than 2 Mb.

**Table 5.** Terpene synthases found in clusters.

Genotype	chr	Start	Stop	TPS	Content
PR	5	0.9 Mb	2.6 Mb	10	3 × TPS10, 3 × Myrcene, 2 × Limonene, 2 × Myrcene
CP	5	1.4 Mb	2.7 Mb	10	3 × TPS10, 3 × Myrcene, 2 × Limonene, 2 × Myrcene
PR	6	80.4 Mb	82.9 Mb	9	2 × Humulene, 4 × Germacrene, 3 × Humulene
CP	6	75.3 Mb	78.3 Mb	10	2 × Humulene, 5 × Germacrene, 3 × Humulene
PR	9	59.2 Mb	59.4 Mb	5	5 × probable monoterpene synthase
CP	9	62.9 Mb	63.0 Mb	4	4 × probable monoterpene synthase

The TPS10 triplet in MTC5 includes one TPS10 and two TPS10-like predictions in both haplotypes.

**Fig. 6.**
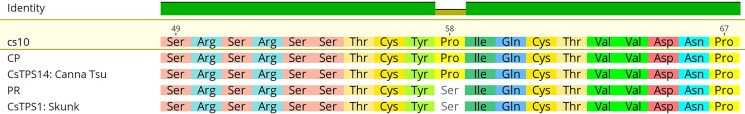
Clustal Omega alignment of limonene synthases from cs10, PR, CP, Canna Tsu, and Skunk.

To corroborate the predicted products, we queried a custom BLAST db, composed of 33 TPS characterized by heterologous expression, with the CDS of TPS found in cs10, PR, and CP. Where a gene contains multiple isoforms, we took isoform X1. To quantify similarity, we report the “Grade”, a proprietary metric within Geneious Prime that incorporates the length, e-value, and percent identity of the hit (Supplementary Table 4). We identified two notable polymorphisms in MTC5. The cs10 gene XP_030500628.1, predicted as “(−)-limonene synthase, chloroplastic like,” was polymorphic, with cs10 and CP having the best (99.8%) hit to CsTPS14: Canna Tsu (−)-limonene, while PR best matched (99.2%) to CsTPS1: Skunk (−)-limonene. Aligning the limonene synthases revealed, among other polymorphisms, a proline-serine transversion shared between PR and Skunk (Fig. 6).

**Fig. 7.**
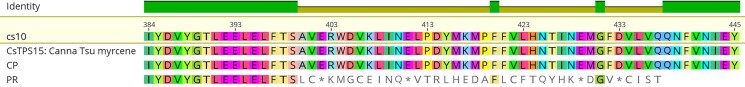
Clustal Omega alignment of myrcene synthases from cs10, PR, CP and Canna Tsu.

Within the same MTC, we also found that the cs10 gene XP_030501051.1, a predicted “myrcene synthase, chloroplastic,” in all cases matched to CsTPS15: Canna Tsu Myrcene; however, the Grade in cs10 and CP was quite good (96.7 and 96.6%), while in PR the Grade was much lower (75.7%). Aligning these synthases revealed several nonsense mutations in the PR allele (Fig. 7).

#### NLRs

We report 227 results in PR and 240 in CP, all of which are placed on the 10 chromosomes. Many of these predictions occur in clusters, which we call Major Resistance Clusters (Christopoulou et al. 2015). Due to their more abundant and diffuse nature, we forego a formal definition and instead rely on a simple visual inspection. Typically, clusters have 5 or more members and an NLR density of at least one NLR per 2 Mb.

In PR, 176 NLRs are found in 9 clusters, and in CP, 188 in 11 clusters, representing 77.5 and 78.3% of the total (Table 6). While most MRC have similar location and copy number between drafts, MRC1a has 8 NLRs in PR compared to just 2 in CP, and MRC5 and MRC7, which contain 4 and 14 NLRs in CP, appear to be absent from PR.

**Table 6.** Location and copy number of major resistance gene clusters.

	PR			CP		
MRC	Start	Stop	NLRs	Start	Stop	NLRs
1a	37,732,869	38,500,894	8	33,106,874	33,180,695	2
1b	65,934,737	87,214,810	18	62,988,874	68,532,952	11
2a	3,093,234	3,150,113	5	1,080,960	11,54,696	2
2b	87,474,025	88,351,507	16	79,836,788	80,842,432	18
3a	39,013	4,472,107	49	8,181	7,630,351	48
3b	76,654,694	85,346,627	20	79,166,000	81,714,877	20
5				80,286,892	80,574,472	4
6a	804,274	9,998,555	30	806,742	10,013,473	35
6b	56,255,482	83,005,153	16	56,615,863	78,421,634	14
7				60,457,601	73,427,240	14
9b	66,855,455	67,250,977	14	69,189,104	71,136,706	20
TOTAL			176			188

#### Repetitive elements

We summarize EDTA and LAI results, and include for comparison EDTA results from the Salk Institute Pangenome (Lynch et al. 2025), which represent the average of 193 assemblies (Table 7).

**Table 7.** Quantification of repeat element composition of Punto Rojo, Cherry Pie, and the average of the Salk Institute assemblies.

	PR	CP	Salk
LINE			
L1	1.56%	2.00%	NR
LTR			
Copia	12.16%	13.72%	16.27%
Gypsy	16.33%	11.62%	19.70%
Unknown	32.53%	35.02%	16.51%
TIR			
CACTA	0.97%	1.31%	3.12%
Mutator	1.97%	2.98%	6.03%
PIF_Harbinger	0.49%	1.25%	1.09%
Tc1_Mariner	0.07%	0.02%	0.37%
hAT	0.97%	0.93%	1.95%
nonTIR			
helitron	1.68%	1.52%	2.84%
repeat_region	2.70%	2.23%	NR
Total	71.41%	72.60%	67.89%
LAI			
Raw	23.70	23.12	NR
Final	18.84	19.22	NR

NR: not reported.

### Comparative genomics

PR and CP were scaffolded to and then aligned against the set of chromosome-scale pseudomolecules shown in Supplementary Table 1. SNPs and larger variants are summarized in Table 8.

**Table 8.** Structural and sequence variation as reported by SyRI for PR and CP.

#Variation_type	PR vs Salk			CP vs Salk			PR vs CP		
	Count	Length_ref	Length_qry	Count	Length_ref	Length_qry	Count	Length_ref	Length_qry
#Structural annotations									
Syntenic regions	2,932	506 Mb	521 Mb	1,295	662 Mb	671 Mb	2,947	488 Mb	493 Mb
Inversions	81	9.50 Mb	9.80 Mb	48	1.93 Mb	2.02 Mb	88	44.7 Mb	48.5 Mb
Translocations	3,208	39.5 Mb	39.4 Mb	866	20.6 Mb	20.5 Mb	3,077	45.6 Mb	45.3 Mb
Duplications (reference)	451	3.7 Mb	–	126	1.3 Mb	–	761	9.45 Mb	–
Duplications (query)	1,568	–	8.0 Mb	1,169	–	7.2 Mb	1,877	–	10.4 Mb
Not aligned (reference)	5,644	176 Mb	–	1,709	48 Mb	–	5,782	219 Mb	–
Not aligned (query)	7,387	–	219 Mb	3,146	–	74 Mb	7,440	–	181 Mb
#Sequence annotations									
SNPs	2,319,292	2.32 Mb	2.32 Mb	2,356,727	2.36 Mb	2.36 Mb	2,527,168	2.53 Mb	2.53 Mb
Insertions	188,373	–	2.37 Mb	242,894	–	1.91 Mb	285,443	–	2.68 Mb
Deletions	290,662	2.99 Mb	–	364,637	2.04 Mb	–	246,441	2.22 Mb	–
Copygains	141	–	0.91 Mb	90	–	0.58 Mb	169	–	2.20 Mb
Copylosses	133	0.55 Mb	–	85	0.53 Mb	–	162	1.70 Mb	–
Highly diverged	38,222	273 Mb	288 Mb	49,826	165 Mb	174 Mb	43,338	317 Mb	325 Mb
Tandem repeats	14	0.01 Mb	0.02 Mb	8	0.00 Mb	0.00 Mb	10	0.02 Mb	0.02 Mb

**Fig. 8.**
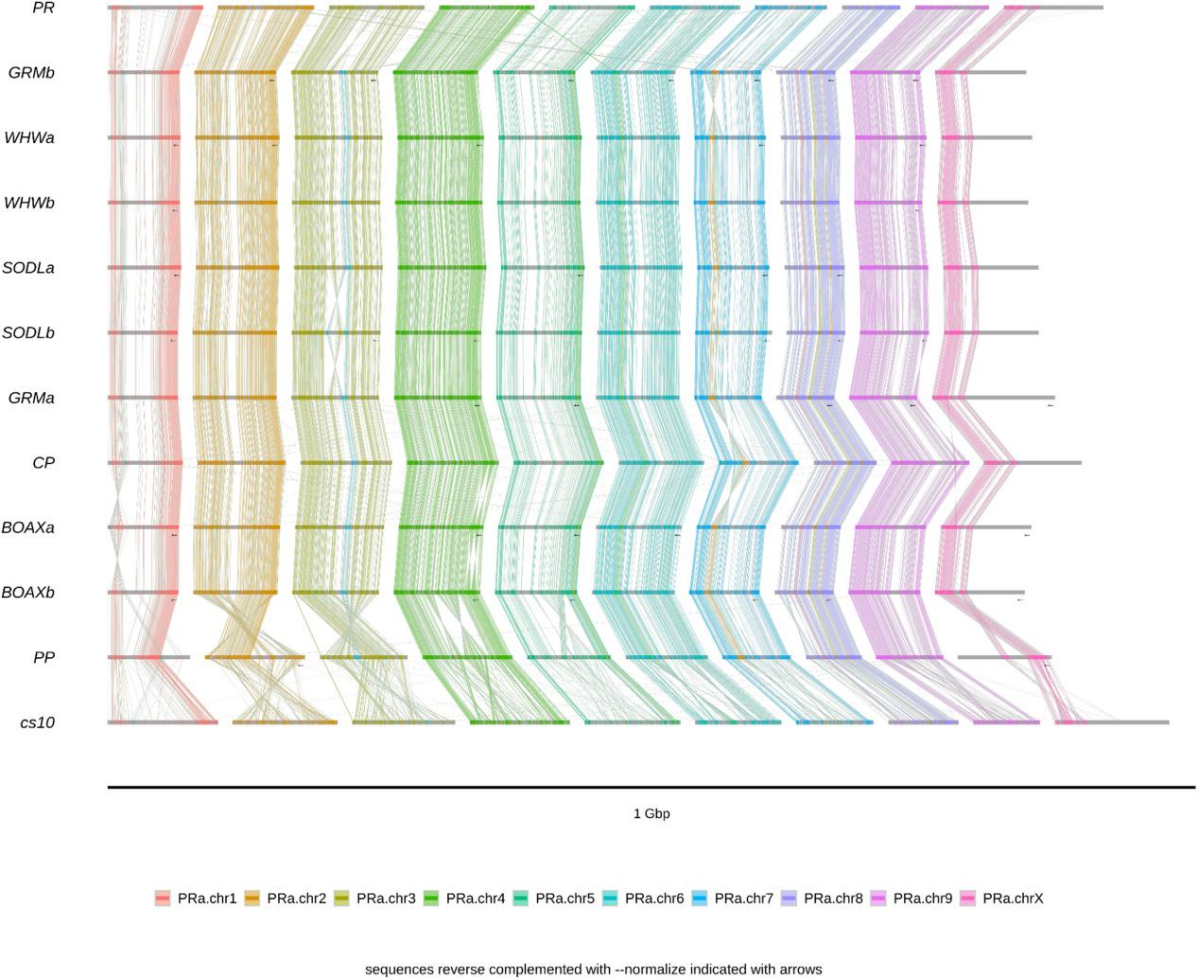
Alignment of 24-mers found in all input assemblies. In addition to PR and CP, we include the genomes whose chromosomes contributed to the scaffolding substrate (Supplementary Table 1), and also Pink Pepper (PP) and cs10, the current and previous NCBI reference assemblies. Black arrows indicate reverse-complemented chromosomes.

To visualize macrosynteny, dotplots were generated for each draft relative to its scaffolding substrate (Supplementary Figs. 2 and 3), and common kmers were visualized with ntSynt (Fig. 8).

**Fig. 9.**
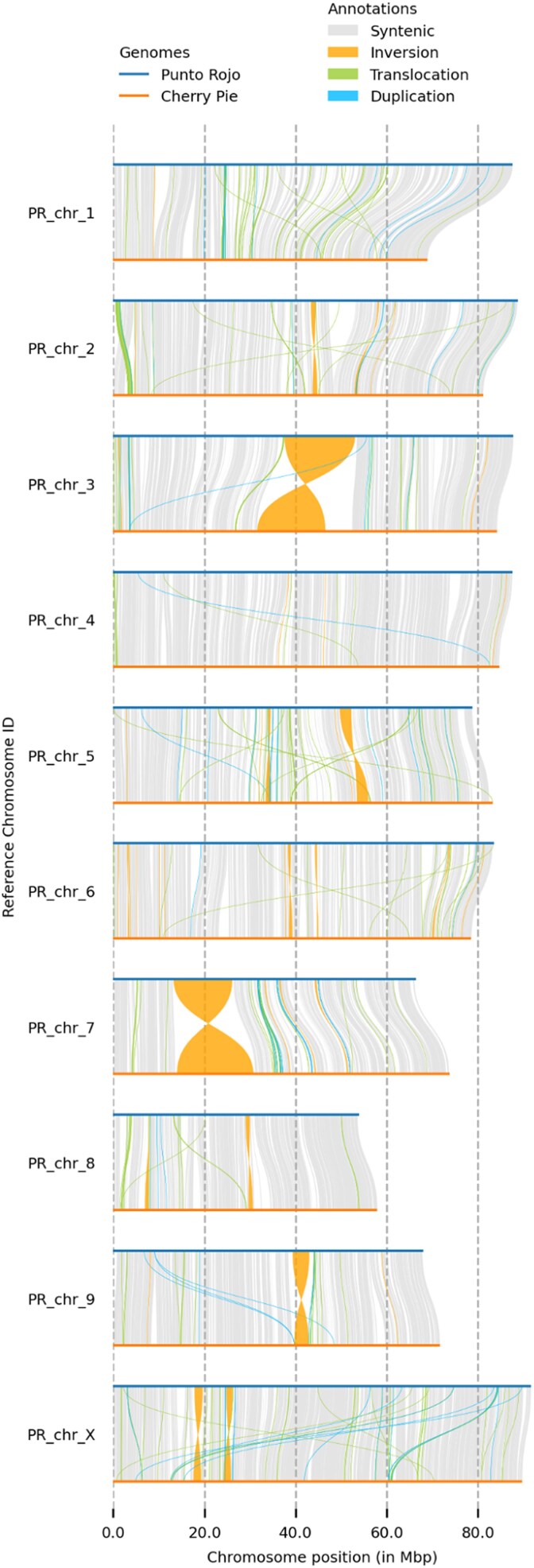
Synteny and rearrangement between PR and CP homologs, filtered above 100 kb.

**Fig. 10.**
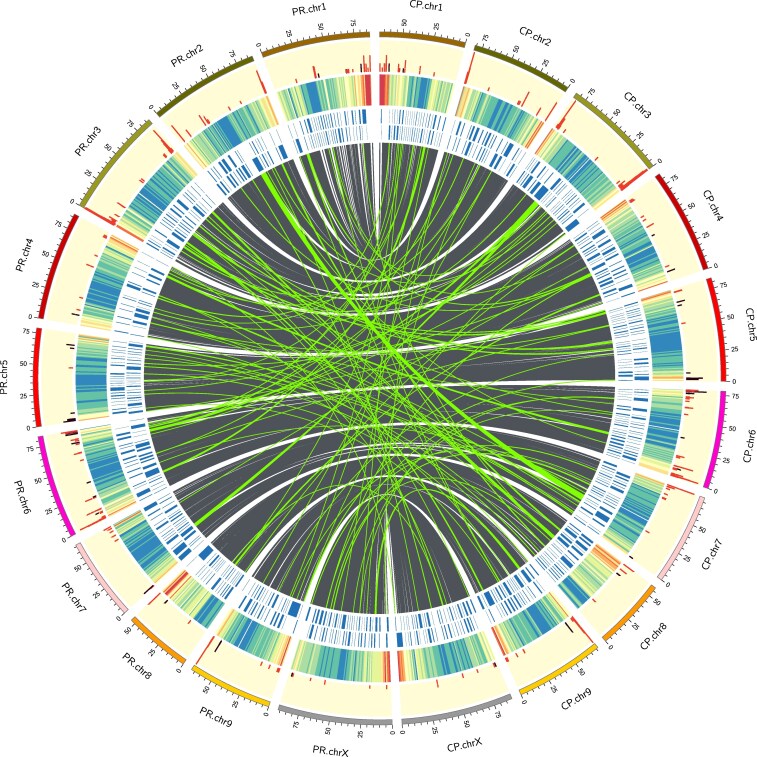
Circos plot showing, from center outwards, homologous regions (grey) and interchromosomal translocations (green), both filtered above 25 kb, contig boundaries (blue), gene density (heatmap, where red is high and blue is low), and NLRs (red) and TPS (purple).

Variation between the two haplotypes was plotted with SyRI and plotsr (Fig. 9), and a Circos plot was generated that, in addition to synteny and interchromosomal translocations, includes tracks for contig boundaries, gene density, and the location of TPS and NLR genes (Fig. 10).

## Discussion

### HMW gDNA prep

Our method produced DNA of adequate length and substandard purity. Given the low yield of 34 Gb, it would be beneficial to refine the technique further, as recent reports indicate that PromethION yields of over 100 Gb are now possible (Belser et al. 2021; van Rengs et al. 2022). Following nuclei isolation, performing the organic extraction with phenol:chloroform (Zerpa-Catanho et al. 2021), in place of mere chloroform, may provide for more efficient removal of carbohydrates and proteins. As well, dark incubation of the shoots for 3d before purification may reduce carbohydrate content (Li et al. 2020).

The decision not to fragment the HMW DNA surely decreased yield, due to accelerated nanopore failure when reading ultra-long fragments (Wang et al. 2021b). However, as the cannabis genome is known to be littered with repeats of 30 to 45 kb (Grassa et al. 2021), the 4 × of ultra-long (>50 kb) coverage found here is likely sufficient to resolve some of the long repeats that might falsely collapse, or fail to extend, in the absence of ultra-long coverage. Therefore, unfragmented DNA appears to be the optimal use of the ONT platform, with the caveat that sequence yield is a function of purity.

### Genome size estimation

Previously, flow cytometry of Cannabis nuclei has reported haploid female estimates of 818 Mb (Sakamoto et al. 1998) and 875 Mb (Faux et al. 2014). Similarly, existing chromosome-scale female assemblies have total base lengths of 714 (Grassa et al. 2021), 796 (Phylos Bioscience, Inc. 2022), 812 (Gao et al. 2020), and 914 (Braich et al. 2020) Mb.

From short read kmers, PR falls into this range, with estimates of 823 and 820 Mb in homozygous and heterozygous mode, respectively, excluding presumed errors. CP, however, gives a size of 43 Mb (hom) and 956 Mb (het), suggesting its kmer distribution is not a good fit for the model. FindGSE and other kmer-based genome size estimators suggest a minimum input of 25 to 30 × (Vurture et al. 2017; Sun et al. 2018), with failures reported at lower coverage (Pflug et al. 2020), and so we assume that the 14.4 × used here was simply inadequate.

We repeated the estimate using the binned, NECAT-corrected long reads. The homozygous estimates of 784 (PR) and 752 (CP) Mb are close to the total contig lengths of 740 and 724 Mb, indicating that this method, which has not been previously reported, appears to provide usable estimates. As these readsets should represent individual haplotypes, it is not unexpected that findGSE failed to complete in heterozygous mode, due to binning having removed the half-size peak that typically permits an estimate of heterozygosity.

### Assembly

#### Trio-binning

Dividing the long reads before assembly has been shown to increase contiguity in both animals (Rice et al. 2020) and plants (Montgomery et al. 2020), but has not previously been reported for Cannabis. In this study, we follow the pattern of the original method, which includes separating reads based on parental 21-mers and discarding the unbinned. As well, we removed 21-mers with homopolymers of length 5 or greater, as these are likely to be erroneous in ONT reads (Wick et al. 2019).

When analyzed for kmer purity in Merqury, every contig is clearly seen to be either maternal or paternal, with no large-scale switches (Fig. 4). However, the SNP-level switch rates of 1.00 and 0.62% are greater than those found in recent reports, based on Hi-C phasing, that return switch rates well under 1% (Kronenberg et al. 2021; Xu et al. 2021; Zhang et al. 2024). These imperfections almost certainly relate to the error rate in the raw reads, which was estimated at 1.8% by comparison with short read kmers. We note that PR received about 5% more sequence than CP, and also produced a more contiguous and gene-complete draft. With R10 ONT reagents providing precision above 99%, future efforts will surely be more accurate.

#### Contiguity

In terms of contiguity and completeness, these results compare favorably to recent assemblies with much higher coverage.

CP and cs10, the initial NCBI reference, are thought to be related as both are CBD clones in the “Cherry” family, which arose in Colorado following legalization in 2012. cs10 gDNA was fragmented to 15 kb, sequenced on the ONT platform to a depth of 100×, basecalled with Guppy 3, assembled in miniasm, polished with Racon-Medaka and Pilon, scaffolded with a Hi-C library, and super-scaffolded at the chromosome scale using a linkage map derived from an unrelated (Skunk × Carmen) F2 population (Grassa et al. 2021).

The contig number, N_50_, and total length of CP are rather similar to cs10, which suggests that longer length and higher accuracy, plus trio-binning, can effectively compensate for lower coverage. In particular, the higher accuracy of Guppy 5 and the good performance of the NECAT assembler, perhaps especially in the error correction phase, appear to allow confident assembly through many repetitive regions with as little as 5 × of coverage. The cannabis genome is known to be littered with repeats of 30 to 45 kb (Grassa et al. 2021), and so the similar N_50_ and contig number may indicate common zones of difficulty that may require additional effort to resolve.

When a Hi-C library is available, scaffolding algorithms frequently break contigs due to uneven coverage. We find this method to be highly variable, with different algorithms finding fewer than 5 or more than 100 putative misassemblies in a draft (Pike et al., in prep). Here, we used Flagger to assess long-read coverage of the contigs, and, apart from contig ends, found just three low-coverage regions, all of which appeared to maintain consistent gene order across them.

#### Completeness

Comparing BUSCO scores of these assemblies to previous reports highlights the value of trio binning. As shown in Table 2, both PR and CP have more single and fewer duplicate single-copy orthologs than the cs10 reference and other published assemblies, and similar numbers of fragmented and missing. By not producing alternate haplotigs, these fully-phased drafts are more frequently able to locate one single-copy ortholog rather than two, which suggests that multicopy paralogs are also likely to be counted more accurately than in pseudohaploid assemblies.

#### Correctness

Merqury estimates the quality of a dual assembly by penalizing kmers found in it that are not found in the corresponding parental dataset. By this metric, PR and CP have quality values (QV) of 24.41 and 24.38, implying base-level accuracy of 99.64 and 99.63%, respectively. This error rate is higher than comparable pseudohaploid drafts built from ONT R9 reads (Read et al. 2020; Belser et al. 2021), which we presume is mainly related to three factors: low yield in our ONT cell, which impacted our ability to ascertain accurate homopolymer lengths, particularly after binning, low coverage of parental short reads, where we had to insist on 1/1 calls (when polishing) to avoid introducing additional switch errors, and the presence of 0.29 and 0.32% other-parent kmers, whose abundance was very close to the implied error rate.

The Earth BioGenome Project, which operates at scale, has proposed that eukaryotic assemblies should have megabase contigs, chromosome-scale scaffolds, Q40 precision, BUSCO over 90%, kmer completeness over 90%, and at least 90% of sequence assigned to chromosomes (Lawniczak et al. 2022). While we admit falling short of the QV standard by more than an order of magnitude, our assemblies well exceed the other parameters (Table 2). As well, the LAI indicates that the LTR content is largely unfragmented (Table 7). Furthermore, given the high degree of macrosynteny observed when compared to several other and more precise assemblies (Fig. 8, Supplementary Figs. 2 and 3), we do not feel that the low QV necessarily implies that major misassemblies are present, only that these assemblies would surely benefit from additional polishing with higher short-read coverage and more accurate long-reads (latest ONT´s R10 pore).

#### Scaffolding

For scaffolding to chromosome scale, ntJoin has been shown to be rapid and precise (Coombe et al. 2020; Wittmeyer et al. 2022). NECAT has been shown to have a very low rate of misassembly (Saud et al. 2021; Wang et al. 2021a), and plant genomes are known to be highly divergent (Goel et al. 2019), so the “no_cut = true” option was used in ntJoin to prevent contigs from being broken when arranged to the heterologous genotype. As well, the “overlap = false” option rescues 140 BUSCO genes that were lost when ntJoin was permitted to merge contigs thought to overlap.

When kmers common to many assemblies are visualized (Fig. 8), a highly conserved architecture is apparent, particularly among the most recent diploid assemblies, such as BOAX, WHW, GRM, and SODL, all built from HiFi reads and scaffolded with Hi-C libraries, which show essentially zero translocations. (We note that this sample includes male and female individuals, resulting in a shortage of common kmers on the X/Y chromosomes.) By comparison, we must admit that the several small translocations seen in PR and CP are likely to be assembly errors, in many cases due to small, repetitive contigs being placed on an incorrect chromosome. While our iterative optimization of the scaffolding substrate reduced this phenomenon, we could not eliminate it.

As well, we acknowledge that our contigs are not able to confirm or deny SVs larger than themselves. For example, the large inversions seen in PR.chr3 and CP.chr7 are inherited from SODLb and GRMb, so that if there are large structural errors in the substrate, our assemblies will merely propagate them. Still, we note that the overall macrosynteny seems good, and while our assemblies do not match the precision of HiFi genomes, their accuracy appears to exceed that of Pink Pepper and cs10, the current and former NCBI references.

#### Diploid assembly

We confirm here the results reported by Nie (Nie et al. 2024), where trio-binning offers, by far, the best phase separation, and PECAT represents a good option for parent-naïve diploid assembly of noisy long reads. Shasta's diploid mode, which the authors acknowledge is “somewhat experimental” (Kolmogorov et al. 2023), offers a draft with very good haplotype resolution, but its small size and low gene completeness render it subpar for downstream analysis. GFAse (Lorig-Roach et al. 2023), which aims to “unzip” linked haplotype bubbles through the use of Hi-C contacts or, as tested here, parental kmers, does more than triple the N_50_ of the draft, but at the expense of many inter-haplotypic joins. We note that the readset analyzed here has N_50_ and coverage about half what is recommended.

### Gene predictions

#### WGS

The placement of 97.3 or 100.2% of genic reference annotations on these two drafts suggests that they are essentially gene-complete. In PR, the total surpasses 100% because Liftoff called copies with 99% or better exonic identity, which resulted in 1,836 genic annotations that appear singly in cs10 and multiply in PR: 852 lncRNA, 849 protein coding, 80 snoRNA, 53 tRNA, and 1 snRNA. In both assemblies, these putative CNVs can be identified by the “copy_number_extra” tag in the GFF. While the cs10 reference is not competitive with new drafts produced since 2020, its annotations, which are based on several RNA-seq datasets as well as a curated set of *ab initio* predictions, provide a solid basis for annotation.

#### CN synthases

The scant corroboration regarding the location of the B locus, and the number of CN synthases found in it illustrates the difficulty of assembling this repetitive region (Supplementary Table 5). Because of the importance of cannabinoids to the Cannabis space, and because of the difficulty in assembling the B locus, it offers a certain parallel with the human major histocompatibility locus, which, due to its importance to human health and “notoriously difficult to assemble” structure (Li et al. 2023), has become a common benchmark for assembly (Gonzalez-Garcia et al. 2023) and variant-calling (Møller et al. 2020) tools. Here, we do not claim our results to be definitive.

All drafts place B on chr7; however, the location varies. PR and Abacus place the active synthase in the range of 50 to 60 Mb, in a cluster with 5 degenerate paralogs, while cs10 and Cannbio-2 place it at 30 to 35 Mb, in a cluster of one active and 10 inactive copies. JL recapitulates the PR/Abacus structure of one active and 5 inactive copies, but places the locus at 90 Mb. CP appears to offer a distinct arrangement, with the active synthase found in a primary cluster abutting 4 degenerate paralogs at about 62 Mb, and a secondary group of presumably inactive synthases with 88 to 89% identity located at 40 Mb.

Given the demonstrated difficulty of assembly, analysis of additional drafts is needed to conclusively resolve what degree of variation at B that is biological and what is technical. The BD and BT alleles have been reported to recombine rarely, if at all (Laverty et al. 2019), despite their high homology, which may be due to one or more large SVs in and around this important locus. In other species, such as corn (Fang et al. 2012) and sunflower (Todesco et al. 2020), massive haplotype blocks have been shown not to recombine, which prevents the separation of alleles that may provide more selective advantage as a group.

#### TPS

With the advent of legalization, several studies (Henry 2017; Orser et al. 2018; Richins et al. 2018) have arrived at the same scheme for classifying the Cannabis population by terpene content: three groups in which the profile, or terptype (Richins et al. 2018), is comprised primarily of myrcene (MYR), terpinolene (TER), or limonene with caryophyllene (LIM). Because these and other terpenes frequently show anxiolytic (do Vale et al. 2002; Ito and Ito 2013; Lima et al. 2013) and antidepressant (Zhang et al. 2019) effects in animal (Wolffenbüttel et al. 2018; Aponso et al. 2020) and human (Haze et al. 2002; Sowndhararajan et al. 2015) models, it is valuable to understand the genetic basis for their accumulation in Cannabis.

Previously, the products of 33 Cannabis terpene synthases had been quantified via heterologous expression in E.coli (Booth et al. 2020). Via BLASTx, we were able to verify many of our gene predictions, clarify others, and also identify promising candidates to be resolved in future investigations. Based on field observations, which corroborate the gray literature, we postulate that PR has a LIM terptype (AllBud.com 2022), and CP a MYR terptype (MrHempFlower.com 2020). Therefore, it is notable that XP_030500628.1, a gene predicted to encode “(−)-limonene synthase, chloroplastic like”, has its best (99.8%) hit in cs10 and CP to CsTPS14: Canna Tsu (−)-Limonene, while in PR it is (99.2%) to CsTPS1: Skunk (−)-limonene. The difference is small, yet a cursory evaluation of an alignment reveals, among other polymorphisms, a proline-serine transversion between the two groups, which indicates that the alleles are in fact distinct (Fig. 6). We also note that XP_030501051.1, a myrcene synthase, is confirmed with best hits to CsTPS15: Canna Tsu Myrcene in PR, CP, and cs10. However, the Grade in cs10 and CP is quite good (96.6 & 96.7%), while in PR it is much lower (75.7%), with a CDS that includes several premature stop codons (Fig. 7). While admittedly scant, these data suggest that the difference between the MYR and LIM terptypes may derive from different numbers of functional myrcene synthases. Because these myrcene and limonene alleles lie within the same MTC on chromosome 5, and gene clusters such as these are frequently co-regulated (Ibn-Salem et al. 2017), analysis of the sequence variation that lies within them is worthy of further inquiry.

We were also able to clarify the role of XP_030484762.1, “probable terpene synthase 9,” which produced a perfect hit to CsTPS29: Blue Cheese Linalool in cs10, and 99.2% hits in PR and CP. Similarly, the 4 TPS on chr9, all predicted as “probable monoterpene synthase MTS1, chloroplastic,” gave near-perfect hits to proteins demonstrated to produce primarily myrcene, terpinolene, or a mix of geraniol and himachalene (Supplementary Table 4).

Several loci did not find good matches among the characterized enzymes. In particular, the (E,E)-geranyllinalool synthases on chr7 and many of the diterpene synthases on chr1 and chr6 had best hits with Grade <80%. Expression in vitro of these types could provide a fuller picture of the terpenes that may modulate the perceived effects of cannabinoids, in what is known as the entourage effect (Russo 2011). For the future, we hope to characterize the three MTC as polygenic Mendelian units, which are likely to be the major contributors to genetic variance in terptype. Because of their position towards the ends of chromosomes (Fig. 10), they are much more likely to be affected by recombination (Grassa et al. 2021), and so may represent some of the fastest evolving regions of the Cannabis genome.

#### NLRs

In rosids, the number of NLRs ranges from 58 to 930 (Ngou et al. 2022), and so the counts reported here (227 and 240) are not atypical. Clustering of NLRs is consistent with the theory that their diversification results from duplication via unequal crossing over, followed by neofunctionalization (Leister 2004). And, the placement of several clusters at the very ends of chromosomes is consistent with their rapid evolution (Lai and Eulgem 2018), especially given that in Cannabis, large central portions of chromosome bodies appear to be insulated against recombination, with most events restricted to their distal ends (Laverty et al. 2019).

NLRs are notoriously difficult to assemble from short reads (Witek et al. 2016), in part due to strong conservation in the NBS domain, with collapsed paralogs and technical chimeras being common artifacts. Sequencing reads of sufficient length to span one or more full-length genes offer more clarity as to cluster structure (Barragan and Weigel 2021), with phasing of haplotypes offering further improvements (Seong et al. 2022). This increased resolution becomes meaningful as trait mapping commences for Cannabis. At present, there is only one R-gene reported, PM1, which confers qualitative dominant resistance to the powdery mildew pathogen *Golovinomyces ambrosiae* (Mihalyov and Garfinkel 2021). While the PR × CP F_1_ has been observed to be susceptible to powdery mildew, accurately assembling this cluster in resistant genotypes will likely be the most efficient path toward elucidating the biochemistry of perception.

**Fig. 11.**
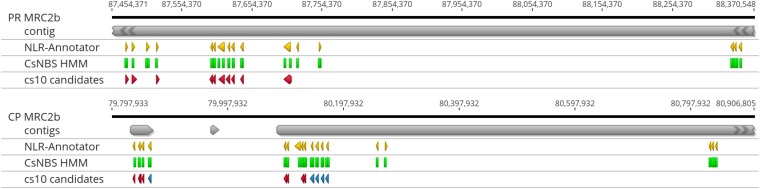
NLR predictions from NLR-Annotator (yellow), the CsNBS HMM (green), and the cs10 reference (red) found in the contigs (grey) that constitute MRC2b in Punto Rojo (top) and Cherry Pie (bottom). In the cs10 track for CP, the 5 homologs of XM030648577.1 are marked in blue, and the best match is at left.

Linkage mapping places PM1 in MRC2b, which contains 16 NLRs in PR and 18 in CP (Table 6). PR assembles the cluster in one contig while CP divides it among three. Visualizing the lifted-over reference annotations, the Cannabis-specific NBS HMM hits, and the NLR-Annotator predictions illustrates the convergence and divergence among callsets (Fig. 11).

The reference predicts 10 NLRs in this region of about 1.5 Mb. In PR, all were present in a single copy. In CP, 8 are present as a single copy; one, XM030647777.1, is absent, while another, XM030648577.1, has four additional copies with exonic identity over 99%. The ordering of these genes varies among PR and CP, and cs10. In both PR and CP, the HMM and NLR-Annotator both predict one additional NLR within the canonical cluster, and 5 additional candidates in the ∼650 kb downstream.

Throughout the genome, we observed that NLR-Annotator made about 40 predictions, mostly under 1 kb, that were not corroborated by the HMM or the cs10 annotations, which we presume to be false positives. The HMM had only a few hits that were not corroborated, but sometimes finds two hits in one gene, particularly on chr2. Therefore, the intersection of the two methods was taken as a parsimonious set.

#### Repetitive elements

With EDTA, we found that PR and CP contained 71.41 and 72.60% repeats, with Copia (12.16 and 13.72%), Gypsy (16.33 and 11.62%), and unknown (32.53 and 35.02%) long terminal repeats (LTR) comprising the largest fractions (Table 7). We note briefly that PR contains more Gypsy than Copia elements, while CP has more Copia than Gypsy; however, given the large unknown fraction, this result must be considered quite preliminary. We suggest, tentatively, that the recent wide hybridization that gave rise to CP may have also activated a burst of Copia transposition, as observed in other plants (Kawakami et al. 2010; Parisod et al. 2010). The Salk Institute Pangenome assemblies, which were also assessed with EDTA, found slightly higher fractions in most categories, with just half the content of unknown LTR (16.51%). The LTR Assembly Indices (LAI) for PR and CP were 18.84 and 19.22, comparable to recent assemblies of Begonia (17.73, Xiao et al. 2025) and Solanum pimpinellifolium (14.49, Han et al. 2024), yet trailing a collection of 26 maize genomes (average of 28, Hufford et al. 2021). A likely explanation for the higher marks in the Cannabis and maize collections is that, by scanning more genomes, fewer elements remain unclassifiable. Therefore, future work might seek to analyze a broader sample of the population in order to leverage intraspecific variability and quantify the repeat content more precisely.

### Comparative genomics

To scaffold the contigs to chromosome scale, a collection of superscaffolds from the recent Salk Institute Cannabis Pangenome was chosen (Supplementary Table 1). These assemblies are assembled from PacBio HiFi reads and scaffolded and phased with Hi-C libraries (Lynch et al. 2025). We observed many fewer small inversions than when scaffolding to other recent long-read assemblies (data not shown), which likely reflects an enhanced ability of Hi-C to properly orient contigs when leveraged against the greater accuracy of HiFi reads. Scaffolding to any one haplotype invariably produced several troubling large-scale rearrangements, with abundant translocations, including contigs being split between two chromosomes. Therefore, a collection of chromosomes was chosen that produced a visually acceptable dotplot, with a minimum of translocations. When PR and CP are aligned to one another (Fig. 9), large inversions can be seen on chromosomes 3, 5, 7, and 9, which are absent when each is aligned to its substrate (Supplementary Fig. 1), and which are not evident when binned, corrected readsets are mapped back to the assemblies. These large inversions may inhibit recombination, as has recently been shown for tomato (van Rengs et al. 2022). However, we must note that, if these inversions are errors in the chromosomes chosen for scaffolding, our assemblies will simply propagate them.

We note that, at the level of SVs, PR appears to be more diverged from its substrate than CP: it shows less synteny (506 vs 662 Mb) and more inversions (9.50 vs 1.93 Mb), translocations (39.5 vs 20.6 Mb), and unaligned regions (176 vs 48 Mb). Meanwhile, the number of SNPs hardly varies (2.32 M vs 2.36 M), highlighting the value of counting larger variants (Table 8). (We note here that SyRI only counts SNPs in syntenic regions.) While scant, these observations suggest that Punto Rojo, a long-flowering landrace of only moderate cannabinoid content, may represent an unusual lineage that remains undersampled among the current crop of Cannabis genomic resources. A large number of anecdotal reports suggest that Colombian landraces were a common founder of modern drug types (Rahn et al. 2016), and so future work should include the sequencing of more Colombian heirlooms, in order to identify characteristic genes or haploblocks that may have persisted in the modern market.

It has been shown that SVs are called more accurately from *de novo* assemblies than from mapping long reads to a reference, especially for variants over 100 kb (Ahsan et al. 2023). SyRI, one of the few tools capable of such an analysis, here shows that PR and CP share 488 Mb (65.9%) of synteny, and have 99.7 Mb of detectable inversions, duplications, and translocations, leaving 219 Mb (29.6%) unalignable. This may seem imprecise when compared to opisthokont genomes that routinely show synteny above 90% (Goel et al. 2019), but more likely reflects the greater intraspecific architectural diversity found in plant genomes, which has only recently become quantifiable, with the benefit of third-generation sequencing. For comparison, when aligning two gold-standard maize genomes (PH207 and B73), SyRI found 62.2% synteny and 32.5% unalignable (Goel et al. 2019). It may be that anemophilous outcrossers are particularly unlikely to purge rare variants, and so in the future we hope that the creation of a complete Cannabis pan-genome can further characterize the structural variation that exists across its range.

## Conclusions

Here, we show that trio-binning can separate noisy ONT R9 reads and produce very good fully-phased assemblies. By avoiding haplotype collapse, we are better able to characterize the content of two important gene classes, which occur in clusters of paralogs, and represent the fastest-evolving regions of the Cannabis genome. We are also able to ascertain the presence of many large structural variants, surpassing the average read length, which are frequently invisible when mapping to a reference.

The natural diversity of Cannabis is remarkable; few species can be found from zero to sixty degrees of latitude and at altitudes from 0 to 3,000 m. Further characterization, including additional genome assemblies and especially multiple genotype, multiple environment field trials, should enlighten as to the variants that facilitate adaptacion to such a wide range of habitat.

The PR and CP parents have both been used to create a wide variety of testcrosses, and we hope that these new assemblies will enable more precise trait mapping than would be possible with an exogenous reference.

## Supplementary Material

jkaf286_Supplementary_Data

## Data Availability

The genomes are available from NCBI under accession codes JBDLLE000000000 (Punto Rojo) and JBDLLD000000000 (Cherry Pie), as part of BioProject PRJNA1090025. The code used to create them is available on GitHub (github.com/COMInterop/PRCP). Additionally, copies named according to PanSN-spec (Garrison 2022), with annotation GFFs, as well as all Supplementary Tables, are available from Zenodo (https://doi.org/10.5281/zenodo.15284085). Supplemental material available at G3 online.
