# Supplementary figures and images for: A trio-binning approach for genome assembly reveals extensive structural variation between two Cannabis cultivars: Punto Rojo and Cherry Pie
Source: G3 (Bethesda). 2025 Dec 30;16(2):jkaf286. doi: 10.1093/g3journal/jkaf286 (PMC12869074; doi:10.1093/g3journal/jkaf286)

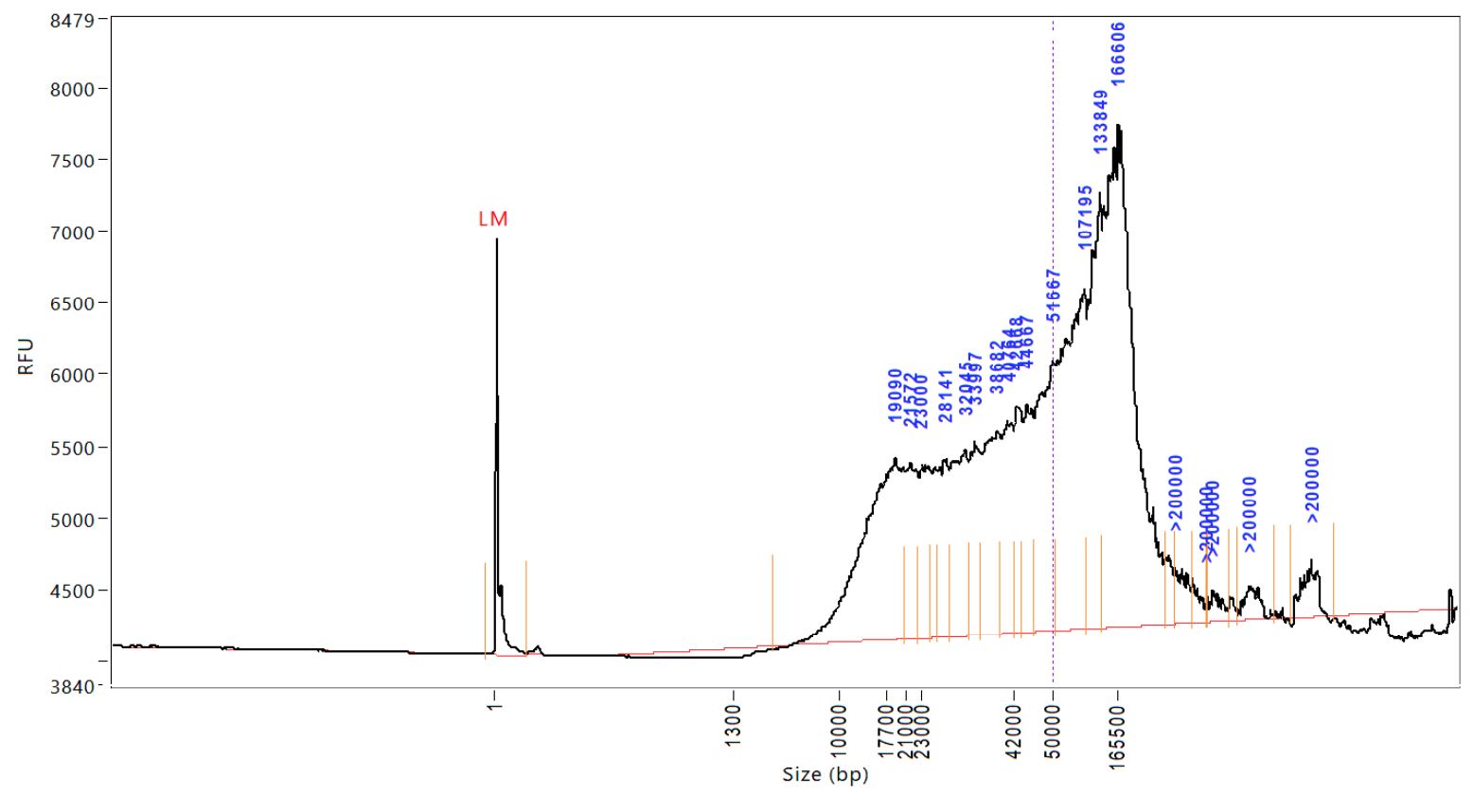

Supplement: jkaf286_Supplementary_Data [file jkaf286_supplementary_data.zip › Supplemental_Figure_1_G3-2025-406342.png]

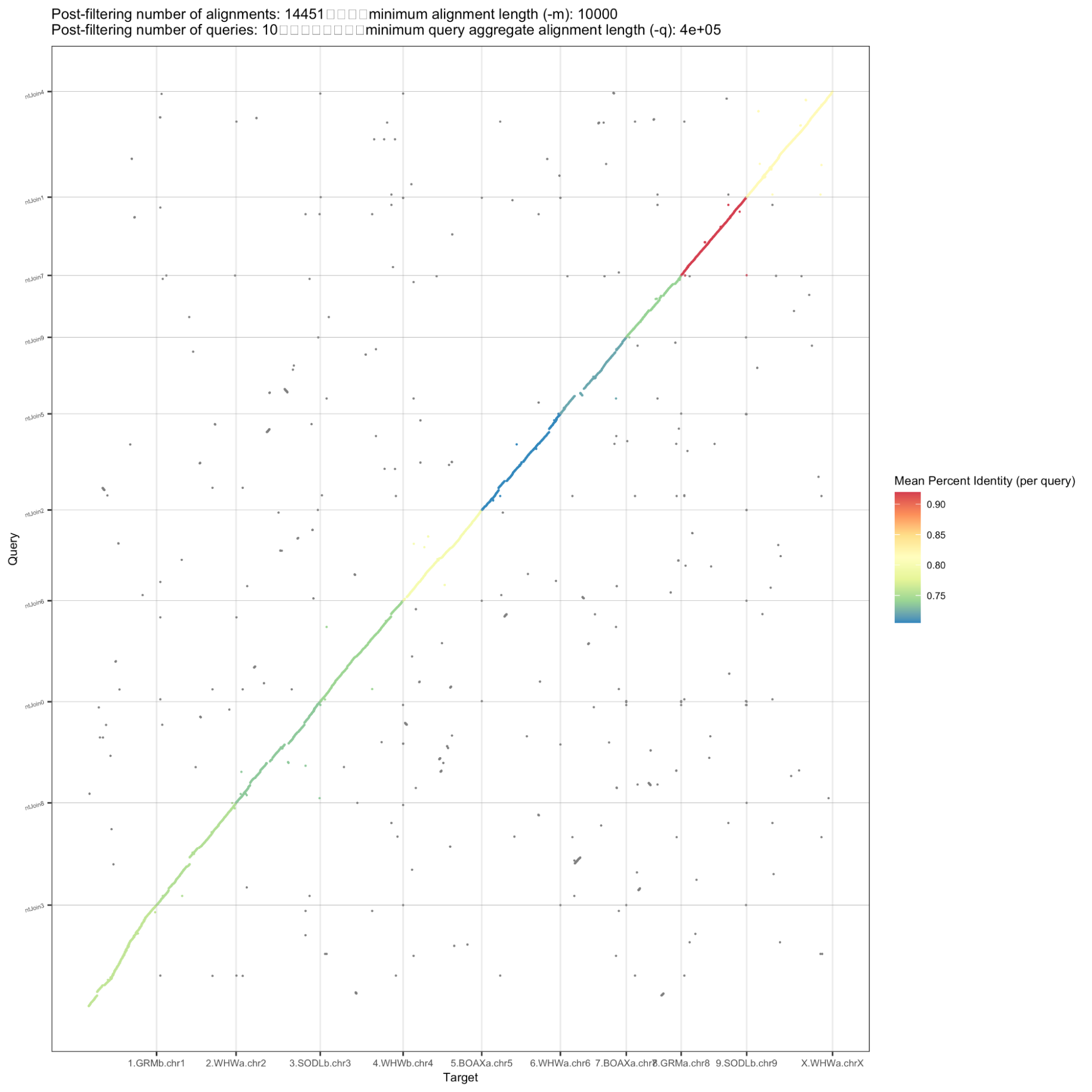

Supplement: jkaf286_Supplementary_Data [file jkaf286_supplementary_data.zip › Supplemental_Figure_2_G3-2025-406342.png]

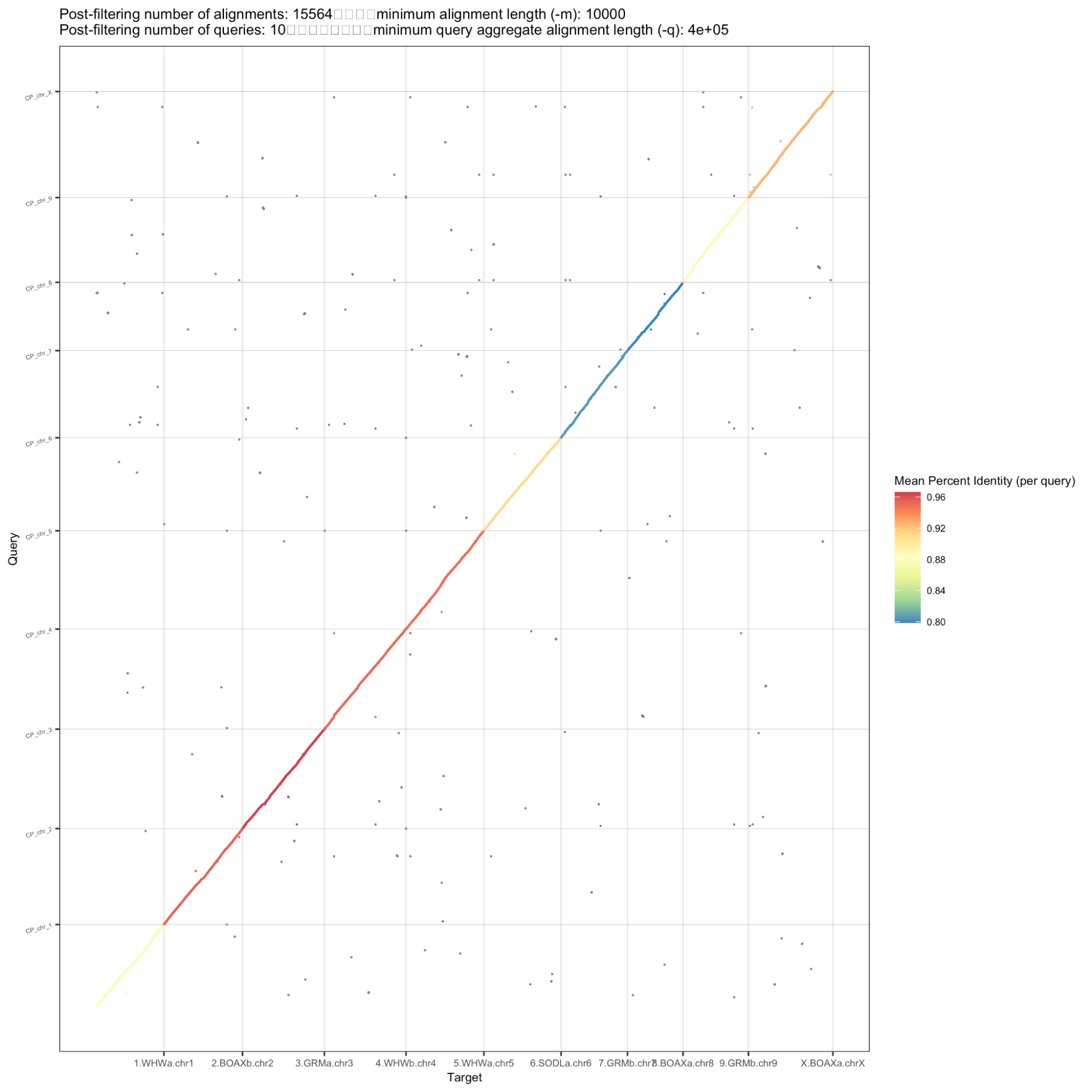

Supplement: jkaf286_Supplementary_Data [file jkaf286_supplementary_data.zip › Supplemental_Figure_3_G3-2025-406342.png]
